# Highly Porous 3D Nanofibrous Scaffold of Polylactic Acid/Polyethylene Glycol/Calcium Phosphate for Bone Regeneration by a Two-Step Solution Blow Spinning (SBS) Facile Route

**DOI:** 10.3390/polym16213041

**Published:** 2024-10-29

**Authors:** Vanderlane Cavalcanti da Silva, Déborah dos Santos Gomes, Eudes Leonan Gomes de Medeiros, Adillys Marcelo da Cunha Santos, Isabela Lemos de Lima, Taciane Pedrosa Rosa, Flaviana Soares Rocha, Leticia de Souza Castro Filice, Gelmires de Araújo Neves, Romualdo Rodrigues Menezes

**Affiliations:** 1 Graduate Program in Materials Science and Engineering, Department of Materials Engineering, Federal University of Campina Grande, Campina Grande 58429-900, Brazil; vanderlane.cavalcanti@estudante.ufcg.edu.br; 2 Laboratory of Materials Technology (LTM), Department of Materials Engineering, Federal University of Campina Grande, Av. Aprígio Veloso 882, Campina Grande 58429-900, Brazil; eudesleonnan@gmail.com (E.L.G.d.M.); gelmires.neves@ufcg.edu.br (G.d.A.N.); 3 Center for Science and Technology in Energy and Sustainability (CETENS), Federal University of Recôncavo of Bahia, Feira de Santana 44042-280, Brazil; adillysmarcelo@gmail.com; 4 Nanobiotechnology Laboratory, Federal University of Uberlandia, Uberlandia 38408-100, Brazil; isabela.lemos@ufu.br (I.L.d.L.); taciane.rosa@hotmail.com (T.P.R.); leticiafilice@gmail.com (L.d.S.C.F.); 5 Department of Oral and Maxillofacial Surgery and Implantology, Federal University of Uberlandia, Uberlandia 38408-100, Brazil; flavianasoares.rocha@gmail.com

**Keywords:** scaffold, solution blow spinning, tissue engineering, nanofiber, hybrid

## Abstract

This work presents the successful production of highly porous 3D nanofibrous hybrid scaffolds of polylactic acid (PLA)/polyethylene glycol (PEG) blends with the incorporation of calcium phosphate (CaP) bioceramics by a facile two-step process using the solution blow spinning (SBS) technique. CaP nanofibers were obtained at two calcium/phosphorus (Ca/P) ratios, 1.67 and 1.1, by SBS and calcination at 1000 °C. They were incorporated in PLA/PEG blends by SBS at 10 and 20 wt% to form 3D hybrid cotton-wool-like scaffolds. Morphological analysis showed that the fibrous scaffolds obtained had a randomly interconnected and highly porous structure. Also, the mean fiber diameter ranged from 408 ± 141 nm to 893 ± 496 nm. Apatite deposited considerably within 14 days in a simulated body fluid (SBF) test for hybrid scaffolds containing a mix of hydroxyapatite (HAp) and tri-calcium phosphate-β (β-TCP) phases. The scaffolds with 20 wt% CaP and a Ca/P ration of 1.1 showed better in vitro bioactivity to induce calcium mineralization for bone regeneration. Cellular tests evidenced that the developed scaffolds can support the osteogenic differentiation and proliferation of pre-osteoblastic MC3T3-E1 cells into mature osteoblasts. The results showed that the developed 3D scaffolds have potential applications for bone tissue engineering.

## 1. Introduction

Scaffolds for tissue engineering must have adequate porosity for cells to migrate and proliferate, allowing for tissue in-growth as well as the efficient mass transport of nutrients, gases, and metabolic waste that satisfies the metabolic needs of tissue engineering [1,2].

Despite the different architectures available, nano-based fibrous scaffolds have garnered significant attention in recent years due to their ability to mimic the extracellular matrix (ECM) [3]. This has been demonstrated in studies on antimicrobial wound dressings [4], scaffolds for cartilage regeneration [5], and tissue engineering applications [6,7]. Since nanofibrous scaffolds have higher porosity, surface area, and surface nano-roughness, which improves cell attachment and proliferation, they are thought of as suitable for bone tissue regeneration [3,8]. There has been notable growth in research focused on the development and application of these materials in bone regeneration [9,10]. Specifically, they have shown potential to enhance the mechanical properties of scaffolds [11,12] and optimize pore size and porosity [13,14], facilitating cell adhesion and proliferation.

However, 3D nanofibrous scaffolds are complex to produce using the current spinning methodologies, especially when dealing with hybrid nanofibrous systems based on polymeric–ceramic scaffolds. This poses a challenge to progress in the field of biomaterials. In hybrid scaffolds, also known as inorganic–organic hybrids, the polymer functions as a matrix that provides structural flexibility and also gives support and protection to the fragile ceramic. On the other hand, the ceramic component, such as calcium phosphates, allows for better control of the degradation of the polymeric matrix, may improve the hydrophilic character, and enhances the bioactivity, since the presence of therapeutic ions helps the modulation of the regenerative process [15,16].

Among the polymers used, polylactic acid (PLA) is looked on as a very useful material for scaffolding; however, despite its good mechanical properties and biocompatibility [17], its hydrophobic character and low rate of degradation impose limitations on the optimization of its final properties [18,19]. These drawbacks can be mitigated by blending PLA with other polymers such as polyethylene glycol (PEG) [20], polycaprolactone (PCL) [21], chitosan (CS) [22], and polyvinyl alcohol (PVA) [23], or by creating hybrid PLA-based composites [24]. PEG is a polymer commonly used to increase the affinity of compounds with low hydrophilicity toward water, in addition to presenting biocompatibility, non-toxicity, and easy elimination from the human body [25,26]. Incorporating PEG into a PLA solution not only facilitates the spinning process due to its plasticizing effect but also alters the structural [27], surface [28], degradation, and mechanical characteristics of the PLA/PEG composite [29]. Studies indicate that the inclusion of PEG in PLA enhances drug release [30] and significantly reduces tumor recurrence [28]. Thus, the PLA/PEG blend is a promising material for producing scaffolds in tissue engineering.

Calcium phosphates (CaPs) are well-known bioceramics and widely used for bone tissue regeneration, as they have excellent biocompatibility, bioactivity, an absence of toxicity, and osteoconductivity [31]. However, CaPs’ fragility limits their applications, and their use in the form of a hybrid is very interesting to combine the positive aspects of polymers and the fundamental contributions of calcium phosphates to the bone regeneration process. Among the different phases of CaPs, hydroxyapatite (HAp) [Ca_10_(PO_4_)_6_OH_2_] and tri-calcium phosphate-β (β-TCP) [β-Ca_3_(PO_4_)_2_] are the most used CaP bioceramics due to their chemical structure that resembles mammalian hard tissues, as well as their biocompatible and bioactive character [31,32]. Particles of these phases have been combined with PLA fibers to improve the bioactivity of the system [33,34]. However, the synthesis of CaP particles inserted in polymeric fibers was very long, up to 48 h, and had low productivity [35]. Moreover, the fine particles produced, particularly the nanostructured ones, tend to easily agglomerate, leading to reduced mechanical strength, composite failure, and decreased potential bioactivity of the final construct. However, recently, CaP nanofibers were produced by SBS [36,37] by a simple and fast route, demonstrating the potential of the technique to produce nanostructured CaPs easily in a morphology that lacks the drawback of particle agglomeration.

Despite advances in the study of CaP/PLA composites and nanofibrous scaffolds, significant limitations persist in current methodologies. These challenges include the difficulty of rapidly and efficiently producing nanostructured CaP/PLA composite fibers, as well as the absence of simpler approaches for the fabrication of three-dimensional nanofibrous scaffolds.

Processing routes such as digital light processing (DLP) [38], fused deposition modeling (FDM) [39], thermally induced phase separation (TIPS) [40], and electrospinning (ES) [41], among others, have been used to produce 3D scaffolds. However, none are effective in the fast production of hybrid scaffolds with adequate porosity, interconnected pores, and high surface area allied with nanofibrous structures suitable for bone regeneration and mimicking the ECM.

On the other hand, solution blow spinning (SBS) stands as a very efficient method for the manufacture of 3D nanofibrous scaffolds from various materials, such as PVA [42], PLA [43], PCL [44], bioglass [45,46], and alumina [47]. Furthermore, this technique offers higher productivity than ES and can be used to rapidly produce nanostructured CaPs [36,37]. Therefore, this study aimed to produce PLA/PEG/CaP nanostructured fibrous scaffolds by the SBS technique and to evaluate their potential for bone tissue regeneration. The effect of the amount and composition of CaP on the characteristics of the scaffolds was studied, as well as the scaffolds’ bioactivity and their ability to promote MC3T3-E1 cell proliferation and viability.

## 2. Experimental Procedure

### 2.1. Materials

Triethyl phosphate (TEP, 99.8%, Sigma-Aldrich, St. Louis, MO, USA) and calcium nitrate (Ca(NO_3_)_2_, 99.0%, Sigma-Aldrich) were used as precursors to produce CaPs. Polyvinylpyrrolidone (PVP, Sigma-Aldrich, M_w_ = 1,300,000 g/mol) was used as a spinning agent, and ethanol (EtOH, Synth, Diadema, Brazil) and distilled water were used as solvents. CaP fibers were produced initially, and then fibrous hybrid scaffolds were manufactured with polylactic acid (PLA, Biomater, Cajamar, Brazil, M_w_ = 75,000 g/mol), polyethylene glycol (PEG, Sigma Aldrich, M_w_ = 8000 g/mol), and dimethyl carbonate (DMC, anhydrous >> 99%, Sigma-Aldrich) as the solvents.

### 2.2. Synthesis of Calcium Phosphate Fibers and Scaffold Manufacture 

**Figure 1:**
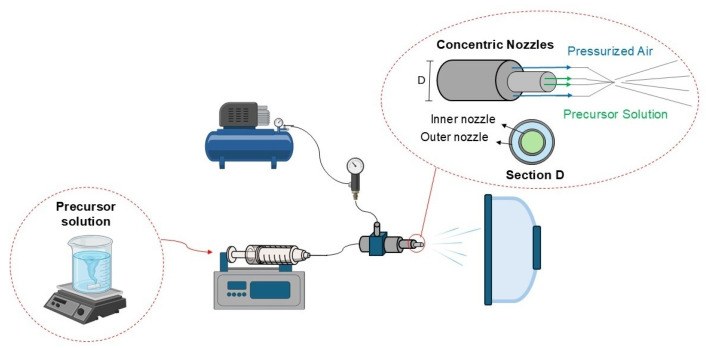
Schematic of solution blow spinning (SBS) process.

Initially, two inorganic precursor solutions with final Ca/P ratios equal to 1.1 and 1.67, respectively, were prepared by mixing Ca(NO_3_)_2_ and TEP in distilled water for an hour, under vigorous stirring, and in capped vials. Meanwhile, two alcoholic solutions of PVP (15 wt%) were separately prepared and further mixed with the aforementioned inorganic precursor solutions to obtain the final spinning precursor solution. The precursor solutions, having different Ca/P ratios, were subjected to the spinning process using the solution blow spinning (SBS) technique, applying a conventional concentric nozzle system described in previous work [36,48]. The whole schematic is shown in Figure 1.

The solutions were force-fed through the inner nozzle at a rate of 6 mL/h, while a high-speed air stream (0.27 MPa) blew across the annulus to facilitate fiber formation. The hybrid fibers traveled across a hollow tube furnace at 80 °C, which accelerates solvent evaporation, towards a static round-shaped collector placed in a heated chamber (40 °C) far from the SBS nozzle, about 80 cm. Finally, the as-spun fibers were calcined at 800, 900, and 1000 °C, at a heating rate of 5 °C/min, for 3 h to remove the organics and to form nanostructured calcium phosphates. The methodology used in this work to produce CaPs using SBS produces CaP nanofibers with the desired compositions in a few hours, including the entire calcination schedule.

To prepare PLA/PEG/CaP hybrid scaffolds, CaP ceramic fibers were gently ground using a mortar and pestle and further dispersed in DMC using magnetic stirring, followed by sonication. Then, PLA and PEG were mixed with the previous CaP/DMC dispersions (15 *m*/*v*%) and stirred at 40 °C for 1 h. Table 1 summarizes the developed formulations and their respective nomenclatures. The final dispersions (PLA/PEG/CaP) were spun into fibrous scaffolds by SBS. The following process parameters were applied: injection rate of 6 mL/h, air pressure of 0.34 MPa, and work distance of 70 cm.

### 2.3. Characterization

#### 2.3.1. Microstructure Characterization

Fiber morphology was assessed on gold-sputtered samples using a scanning electron microscope (SEM) (Shimadzu, SSX-550, Kyoto, Japan). Fiber diameters were measured by ImageJ (Version 1.48, NIH), using at least 200 individual fibers per sample across two different sites.

The thermal behavior of spun CaP fibers and hybrid scaffolds was evaluated by recording the weight loss in a DTG-60 (Shimadzu) apparatus, using approximately 5 mg samples held in platina crucibles. The set was heated from room temperature to 700 °C under an oxidative atmosphere (synthetic air, 21% O_2_ + 79 N_2_) at a heating rate of 15 °C/min. 

The samples were analyzed by transmission electron microscopy (TEM) (FEI Tecnai, G2 F20, Twin, FEI Company, Hillsboro, OR, USA) and the structure was evaluated by selected area electron diffraction patterns (SAED). The crystalline structure of the samples was determined by X-ray diffraction, XRD (Shimadzu). A Ni-filtered Cu Kα radiation source was used (λ = 1.5418 Å) at 40 KV, 30 mA, and 0.02° per minute with 2θ.

The apparent porosity of the scaffolds was measured by the Archimedes method using distilled water as the liquid medium.

To determine the static contact angle, a 5 µL drop of deionized water was carefully deposited onto the surface of the scaffolds. Images were captured using a Canon EOS REBEL T3i camera (Canon, Tokyo, Japan) mounted on a tripod with backlight for better definition. Measurements were performed under controlled temperature (25 °C) and humidity conditions. The contact angles, both left and right, at the liquid–solid–air interface were determined using ImageJ software for image analysis.

#### 2.3.2. In Vitro Apatite Formation

The scaffolds were immersed in a simulated body fluid, SBF (0.5 mg·mL^−1^ in capped vials), prepared according to the literature [49]. The samples were kept in an orbital shaker at 37 °C for periods of 7 and 14 days. Later, the samples were collected, washed with deionized water, and dried in a desiccator at room temperature before SEM investigation.

#### 2.3.3. Cell Viability Assay 

MC3T3-E1 cells were cultured in Minimal Essential Medium (MEM) supplemented with 10% FBS, penicillin (100 U/mL), and streptomycin sulfate (100 U/mL) in a humidified atmosphere of 5% CO_2_ in air at 37 °C. Cells were fed with fresh media every two days until the cells reached near confluence (75%–85%) and trypsinized using trypsin for the next passage. For cell seeding, fiber scaffolds were sterilized in a 70% ethanol solution overnight and exposed to UV light for 1 h. For osteogenic differentiation assays, the culture medium was changed into osteogenic induction media supplemented with 10% FBS, 10 mM β-glycerophosphate, and 50 µM L-ascorbic acid 2-phosphate (Sigma-Aldrich^®^, St. Louis, MO, USA). 

#### 2.3.4. Cell Proliferation Assay

The cell proliferation was evaluated according to the MTT method. For the experiments, cells were seeded on each scaffold at a density of 3 × 10^4^ cells/well in 96-well tissue culture plates and incubated for 7, 14, and 21 days. After that, MTT dye solution (10 µL, 5 mg/mL) was added to each well at the predetermined time point. MTT was aspirated, and 100 µL of dimethyl sulfoxide (DMSO) (Sigma-Aldrich^®^, St. Louis, MO, USA) was added after 4 h of cultivation. Then, 100 µL of the solution was added to another 96-well plate for the absorbance measurement at 540 nm using a microplate reader (Thermo Scientific Multiskan GO Microplate Spectrophotometer^®^, Fisher Scientific Oy, Vantaa, Finland).

#### 2.3.5. Total Protein Dosage

The total protein content of MC3T3-E1 cells was quantified by the Lowry method modified after 7, 14, and 21 days in culture. The samples were rinsed with 0.1 M phosphate buffer at 37 °C and treated with 2 mL of 0.1% sodium lauryl sulfate for 30 min. After that time, 1 mL of this solution was added to 1 mL of Lowry solution and incubated at room temperature for 20 min. Subsequently, 0.5 mL of the Folin–Ciocalteu phenol reagent (Sigma-Aldrich^®^, St. Louis, MO, USA) was added, and the samples were incubated at room temperature for another 30 min. The reading was performed in a spectrophotometer (Ultrospec 1100 pro UV/visible spectrophotometer 200–900 nm, incl. DPU-414 Thermal Printer^®^, Amersham Biosciences, Waukesha, WI, USA) at 660 nm.

#### 2.3.6. Quantification of Alkaline Phosphatase (ALP)

The ALP activity of MC3T3-E1 cells was determined to evaluate osteogenic differentiation in the scaffolds. The samples were rinsed with PBS, and 200 μL of 1% sodium lauryl sulfate was added to each well, which was left to stand 40 min. Then, they were centrifuged for 15 min (1500 G), and the supernatant was collected for analysis. ALP activity was quantified according to the recommendations of the manufacturer of the Alkaline Phosphatase Assay Kit (Labtest^®^ kit, Labtest Diagnóstica, Lagoa Santa, MG, Brazil) at 7, 14, and 21 days. After processing, the samples were read on a spectrophotometer (Ultrospec 1100 Pro—Amershan Biosciences) at 590 nm.

#### 2.3.7. Statistical Analysis 

A *t*-test and *F*-test at 5% significance were used to evaluate the mean fiber diameter and the variance, respectively. All biological experiments were replicated three times, and the analysis was performed using statistical software (GraphPad Prism version 5.0 for Windows, San Diego, CA, USA). The results are reported as mean ± standard deviation (SD), and the differences between groups were determined using ANOVA (the Bonferroni method was used for correction with multiple comparisons). Differences were considered statistically significant when *p* < 0.05.

## 3. Results and Discussion

**Figure 2:**
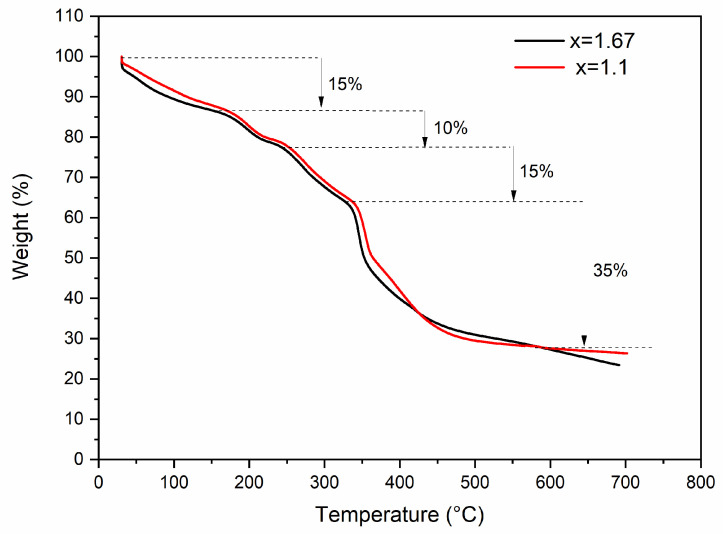
Thermogravimetric analysis of calcium phosphate precursor fibers before calcination produced by SBS for two different Ca/P ratios: 1.67 and 1.1.

Figure 2 shows the thermal behavior of the as-spun fibers with different Ca/P ratios. They were quite similar, since the same precursors were used, and consisted of four thermal events, as follows: residual water and organic solvents evaporate within the first thermal event from room temperature to 170 °C, giving a weight loss of approximately 15%wt; a second weight loss of 10%wt between 170 and 255 °C is related to the decomposition of the inorganic precursors; and 15%wt loss (255–350 °C) can be attributed to residuals of precursors and the onset of PVP degradation through removal of the side groups. Finally, an approximately 35%wt loss due to degradation of the PVP main chain and complete decomposition happens from 350 to 600 °C. The remaining inorganic material obtained at 600 °C was approximately 25%wt for both Ca/P ratios. This small change in thermal behavior between the curves of fibers composed of Ca/P = 1.63 and Ca/P = 1.1 may be related to both phases being composed of calcium and phosphorus, sharing similar crystal structures, resulting in similar thermal events. Similar decomposition events were found in the literature for HA fibers when PVP was used as a spinning aid [36,37,50].

**Figure 3:**
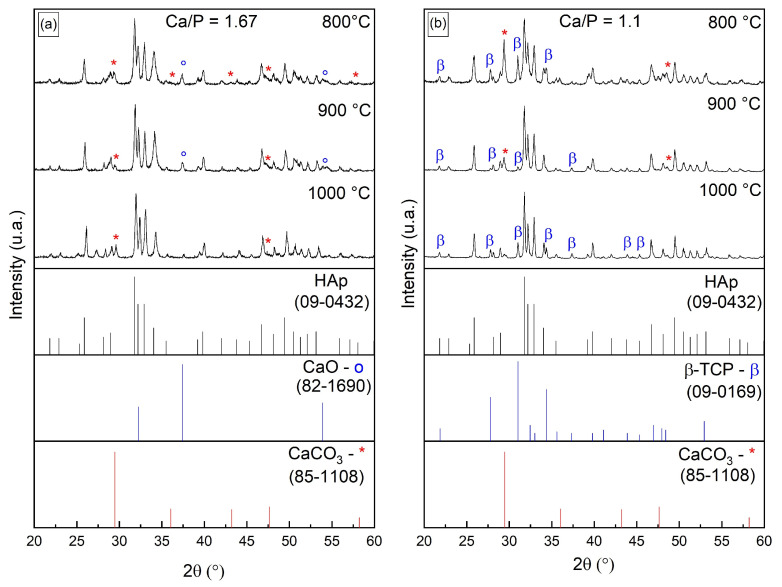
X-ray diffraction patterns of calcined CaP fibers with Ca/P ratios of (**a**) 1.67 and (**b**) 1.1.

XRD patterns of calcined CaP fibers are shown in Figure 3. HAp was the major phase formed regardless of Ca/P ratio and temperature protocol employed, which is corroborated by the intense diffraction peaks at 2θ values of 25.2°, 37.8°, 48.0°, 53.9°, 55.2°, 62.6°, and 68.7° (JCPDS 09-0432), similarly to that found in other studies [51,52,53]. HAp peaks increased and became sharper with increases in temperature, indicating crystallinity augmentation [54].

Beta tri-calcium phosphate (β-TCP) was formed when the Ca/P ratio was 1.1 (peaks at 2θ values of 21.7°, 27.7°, 30.9°, 34.5°, 37.3°, 43.9°, and 45.3°, JCPDS 09-0169) as a result of the increment in the phosphorus content. The formation of β-TCP occurs when the Ca/P ratio is below 1.67, that is, in calcium-deficient phosphate compositions precursors as compared to stoichiometric hydroxyapatite (Ca/P = 1.67) [31,55]. When the Ca/P ratio is adjusted to 1.1, there is an excess of phosphate ions relative to calcium ions, which favors the formation of β-TCP, which also has a distinct crystal structure and lower calcium content [55,56].

The incomplete reaction between the reactants rendered two other phases: calcium carbonate (CaCO_3_) in both Ca/P ratios, as evidenced by peaks at 2θ values of 29.3°, 35.9°, 42.9°, 47.2°, 48.6°, and 58.0° (JCDPS 85-1108), and calcium oxide (CaO) at a CaP ratio of 1.67 (peaks at 2θ values of 37.32° and 54.38°, JCPDS 82-1690). Formation of CaO is favored when the reaction between triethyl phosphate (TEP) and calcium nitrate is incomplete. The slow reaction between the precursors requires extended stirring time to fully form P-O-Ca bonds. CaO was not detected at a Ca/P ratio of 1.1 due to the lower amount of calcium (and calcium precursor) in the composition, which required less time for dispersion, homogenization in the solution, and consequently for reaction with TEP. The higher amount of ‘free’ calcium at a Ca/P ratio of 1.67 enables the formation of CaO and CaCO_3_ due to incomplete carbonation of the material. Over time, all the CaO is likely to carbonate and become CaCO_3_. This behavior was also found by Natarajan and Rajeswari [57] and Eshtiagh-Hosseini et al. [58] when studying the influence of varying the inorganic precursors’ final composition. Since these unwanted phases considerably reduced with the increase in calcination temperature, as judged by XRD, the temperature of 1000 °C was chosen for the manufacture of hybrid scaffolds.

**Figure 4:**
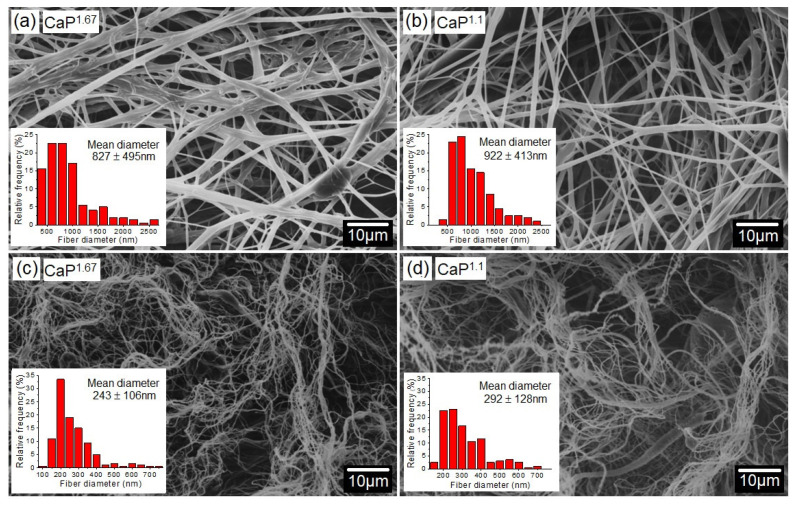
SEM micrographs and fiber distribution of as-spun fibers (**a**,**b**) and CaP fibers calcined at 1000 °C (**c**,**d**).

The microscopic aspect of the spun fibers was typical of cotton-wool-like structures. The SEM micrographs of as-spun (Figure 4a and Figure 4b, respectively) and calcined CaP (Figure 4c and Figure 4d, respectively) fibers showed randomly aligned non-woven fibers. Some were conjoined due to the lack of complete solvent evaporation during the spinning process. This behavior was also observed by Santos et al. (2018) [59] and Oliveira et al. (2019) [36], who observed that incomplete solvent evaporation causes coalescence of fibers. After the thermal treatment at 1000 °C, the mean diameter of as-spun fibers reduced from 827 ± 495 nm and 922 ± 413 nm for Ca/P ratios of 1.67 and 1.1, respectively, to 243 ± 106 nm and 292 ± 128 nm. This resulted from the removal of organics, crystallization, and densification of ceramic phosphates. Studies on ceramic fibers produced by SBS also showed a significant decrease in fiber diameter after the calcination process, due to the removal of polymers used as spin agents as well as owing to the loss of mass from the precursors [36,48,60].

**Figure 5:**
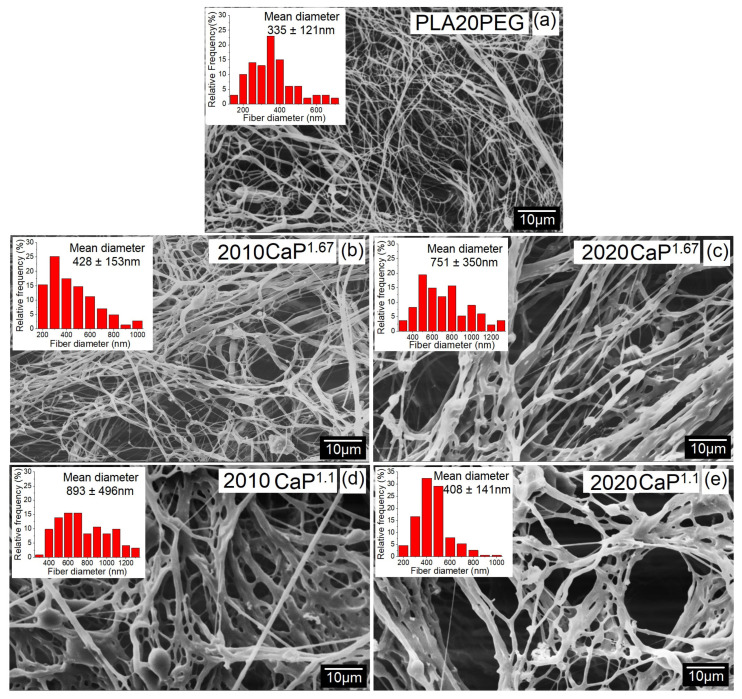
SEM micrographs and fiber diameter distribution of the scaffolds produced by SBS: (**a**) PLA20PEG, (**b**) 2010CaP^1.67^, (**c**) 2020CaP^1.67^, (**d**) 2010CaP^1.1^, and (**e**) 2020CaP^1.1^.

Figure 5 shows the SEM micrographs of the scaffolds obtained. Hybrid scaffolds displayed a morphology comprising a non-aligned bunch of fibers with a randomly interconnected and highly porous structure. Also, the scaffolds showed significant interfibrillar space, which is of fundamental importance for cell migration and fluid transfer in a system used as a scaffold. The addition of calcium phosphate promoted an increase in the fiber diameter when compared to the scaffold without CaP (*p* < 0.05), as shown in Table 2. The diameter values found are in the range of bone collagen fibers (100–2000 nm) [61], which indicates a structural similarity between the produced scaffolds and bone growth environments. 

**Figure 6:**
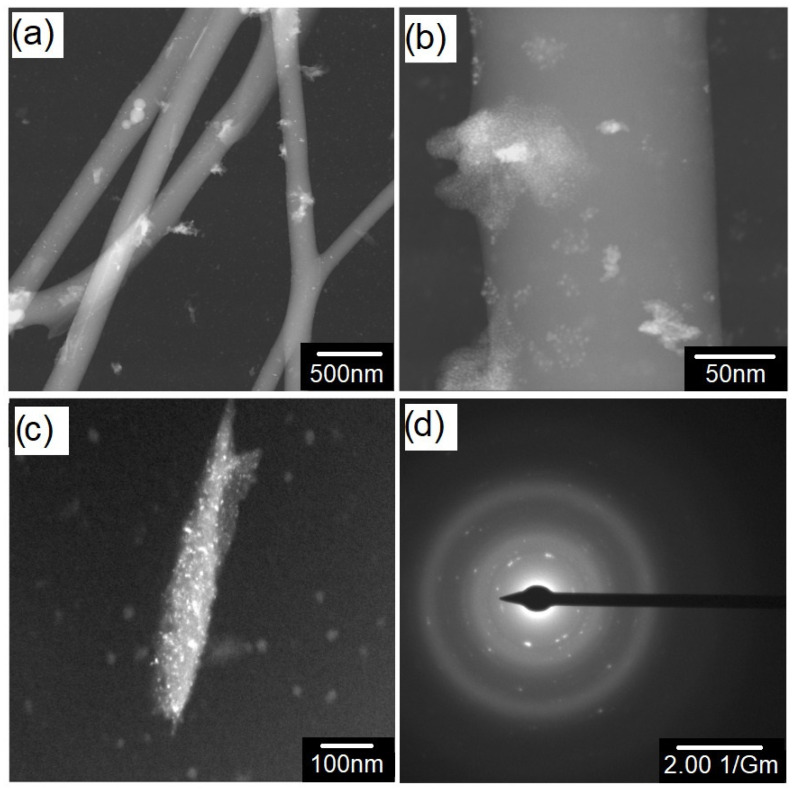
(**a**–**c**) Dark-field TEM micrographs of 2020CaP^1.1^ and (**d**) SAED pattern of the selected area for the same scaffold.

Scaffolds produced with CaP^1.1^ have a reduction in the amount of beads (Figure 5) and a decrease in mean diameter with the rise in the amount of CaP (*p* < 0.05) (Table 2). The scaffolds with CaP^1.67^ presented thicker hybrid fibers with the increase in the amount of CaP (*p* < 0.05). However, for both Ca/P ratios, the CaPs added to the composition were inserted into the PLA/PEG fibers, as shown in detail in the TEM images (Figure 6). The morphology shown in the SEM images (Figure 5) corroborates the TEM images presented in Figure 6 for the produced scaffolds. CaP nanofibers after insertion in the PLA/PEG blend were transformed into whiskers/small rods and particles dispersed along the fibers, ranging in size from 10 to 50 nm. Despite the use of a high percentage of CaP (20%), the particles were incorporated into the fibers with minimal agglomeration, which may be attributed to their hydrophobic nature [62]. 

The randomly arranged nature of the whiskers/small rods and particles indicates effective dispersion within the polymeric fibers and confirms the successful incorporation of CaPs into the nanofibers. Furthermore, this incorporation, along with the combination of inorganic and organic phases at the nanoscale to submicron scale, may enhance the overall performance and increase the mechanical strength.

The SAED result (Figure 6d) consisted of halos and a few diffraction dots. The halos indicate the predominant amorphous nature of the PLA matrix after the SBS process, as described by Santos et al. (2018) [59]. The diffraction dots indicate the crystalline character of CaP particles dispersed along the fiber.

The average fiber diameters obtained for the hybrid fibers are similar to those observed in the literature for electrospun PLA/HAp nanofibers, which range from 215 to 1170 nm [63,64]. However, the distribution of fiber diameters was larger than those observed in the literature for electrospun (ES) fibers. The diameters of fibers produced by SBS are generally slightly larger than the diameters obtained by ES under similar spinning conditions. 

Studies observed that it is a challenge to uniformly disperse HAp powders into PLA. Nanoparticle agglomeration limits the chemical function of a material and makes it difficult to spin fibers [65]. Moreover, big agglomerates impacted the morphology and uniformity of PLA fibers [63] and were capable of changing the nanostructured character of the 3D morphology. Most procedures used to obtain HAp, including commercial ones, employ precipitation routes capable of rendering particle sizes lower than 50 nm, which poses challenges in the dispersion of the individual particles in the polymeric matrix towards the maintenance of suitable mechanical properties [66]. Also, these agglomerates will impact the desired surface nano-roughness of the fiber and the homogeneous distribution of HAp in the fibers, decreasing the potential for cell adhesion and proliferation.

The scaffolds obtained presented high apparent porosity, with values of 88.6% for 2010^1.67^ and 92.4% for 2010^1.1^, characteristics that are essential for their use in biomedical applications, especially for the production of scaffolds for tissue engineering. High porosity is essential to promote cellular infiltration, migration, and vascularization, as well as the flow of nutrients and oxygen, in addition to facilitating the removal of cellular metabolic waste. These attributes are widely recognized in the literature as critical for the success of porous materials in biological environments, favoring tissue regeneration and integration with the host tissue [1,67].

**Figure 7:**
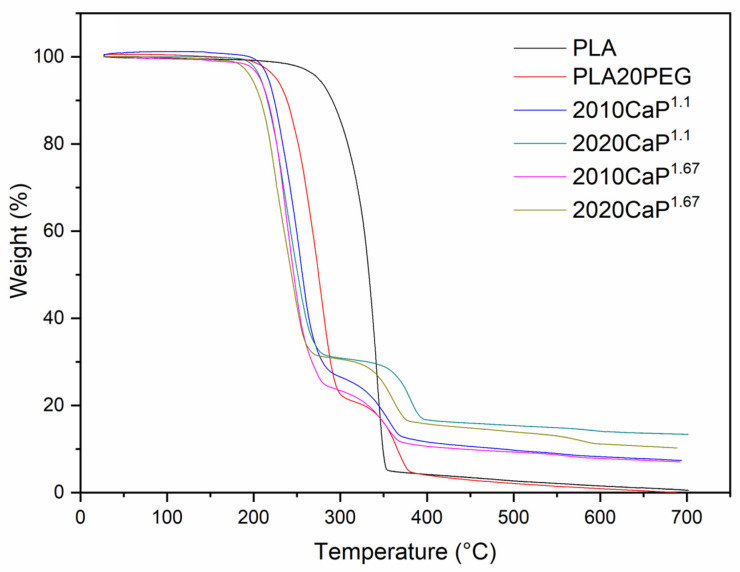
Thermogravimetric analysis of the scaffolds produced by SBS with 20 % PEG.

The amount of Ca/P incorporated into the hybrid scaffolds and their stability were assessed by thermogravimetric analysis (Figure 7). The bioactive filler contents ranged from 10 to 20 wt%, close to those designed in the spinning process.

Fibers of neat PLA decompose between 230 and 350 °C in a single step (degradation of the terminal groups of the main chain or changes in the ester group [68]). The polymer blend-based scaffolds showed a two-step degradation process, as expected: the first corresponds to the PLA degradation, and the second thermal event is regarded as PEG decomposition [69]. Also, the figure shows that the scaffolds remained thermally stable up to 180 °C. However, their stability decreased with the addition of PEG, as evidenced by the decrease in the onset temperature of thermal decomposition. Similarly, the addition of CaPs led to a change in the onset temperature for thermal decomposition from 230 °C (pure PLA) to 190 °C and 170 °C for PLA20PEG and 2020^1.67^, respectively. PEG acts as a plasticizer and makes PLA more thermally sensitive, which decreases the decomposition temperature [70,71]. Some authors [72,73] found that the presence of CaP phases in PLA reduces the enthalpies of crystallization and melting, decreasing the stability of the material. Thus, the observed decrease in PLA stability can be related to these phenomena and will be reflected in the form of a higher degradation rate of the developed systems in the human body environment.

**Figure 8:**
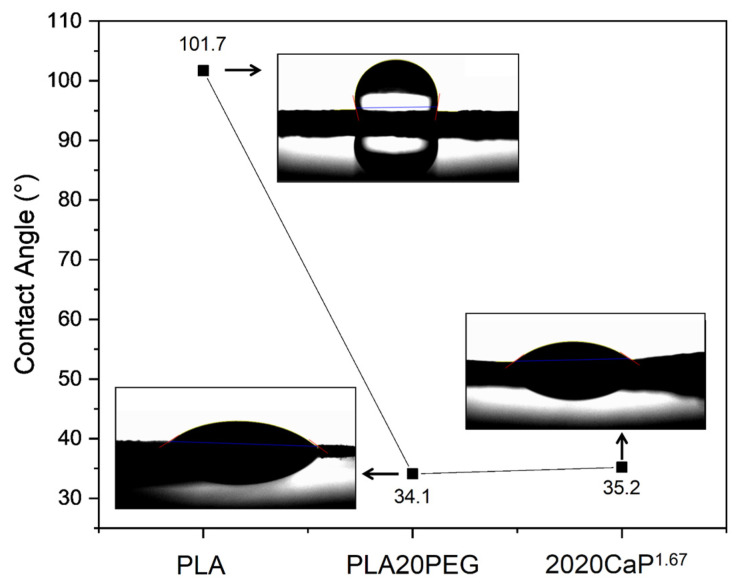
Contact angle of PLA, PLA20PEG and 2020CaP^1.67^ scaffolds produced by SBS technique.

The scaffold formed by PLA (Figure 8) showed a high contact angle of approximately 101.7 ± 1.8°, which is expected due to PLA’s hydrophobic nature. High contact angles (90° < θ ≤ 180°) are typical of materials with low wettability [43,74]. Scaffolds with high hydrophobicity tend to promote low cell adhesion, which can potentially lead to inflammatory responses [75,76]. When adding PEG up to 20%wt, a significant reduction in the contact angle values was observed, indicating an increase in wettability. Specifically, the PLA20PEG scaffolds presented a contact angle of approximately 34.1 ± 0.3°, while the scaffolds containing 2020CaP^1.67^ presented a slightly higher value, around 35.2 ± 0.1°. The contact angle of the scaffolds with PEG was below 90°, demonstrating that the presence of this polymer conferred hydrophilicity to the material, making it more soluble in water due to the presence of many hydrophilic hydroxyl groups in its structure [77].

**Figure 9:**
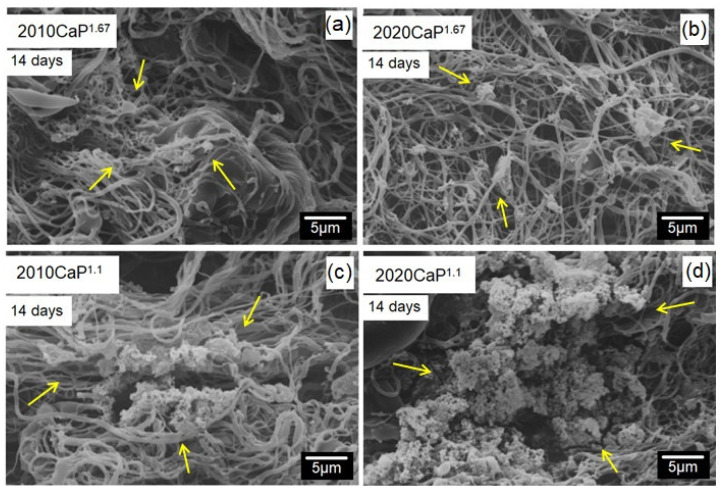
SEM micrographs of the PLA/PEG/CaP scaffolds submitted to SBF for 14 days with Ca/P ratios of 1.67 (**a**,**b**) and 1.1 (**c**,**d**). The yellow arrows indicate the deposition of the typical apatite structure.

The biocompatibility of the scaffolds was investigated in vitro by SBF assay, and the results are shown in Figure 9. Little or no deposition of apatite took place within 7 days of immersion in SBF. However, a typical cauliflower-like apatite structure was deposited on the surface of the scaffold fibers as the immersion time increased. This deposition is indicated by yellow arrows in Figure 9. PEG has the ability to increase the aqueous solubility and dissolution characteristics of scaffolds, influencing its mineralization rate [28,78]. Also, the amount of apatite deposited was higher at the ratio of CaP^1.1^ (Figure 9c,d). This might be related to the fact that the presence of the β-TCP phase, of higher solubility as compared to HAp, exhibited a rapid release of osteogenic ions and favored apatite formation [79,80]. β-TCP and HAp have different characteristics, i.e., solubility, density, crystallinity, etc., which implies different interactions with the in vivo environment [81,82]. β-TCP’s resorption rate depends on its porosity; it is 10 to 20 times faster than HAp [83], and it can be resorbed between 6 and 15 weeks after implantation. The mixture of these materials is clinically viable for applications in bone substitutes [84]. The presence of the more soluble calcium phosphate (β-TCP) increases the concentration of calcium and phosphate ions near the fiber surface, creating a suitable environment for the formation of the apatite layer [84,85]. This layer provides an appropriate environment for subsequent steps, such as bone cell proliferation and differentiation [86,87].

**Figure 10:**
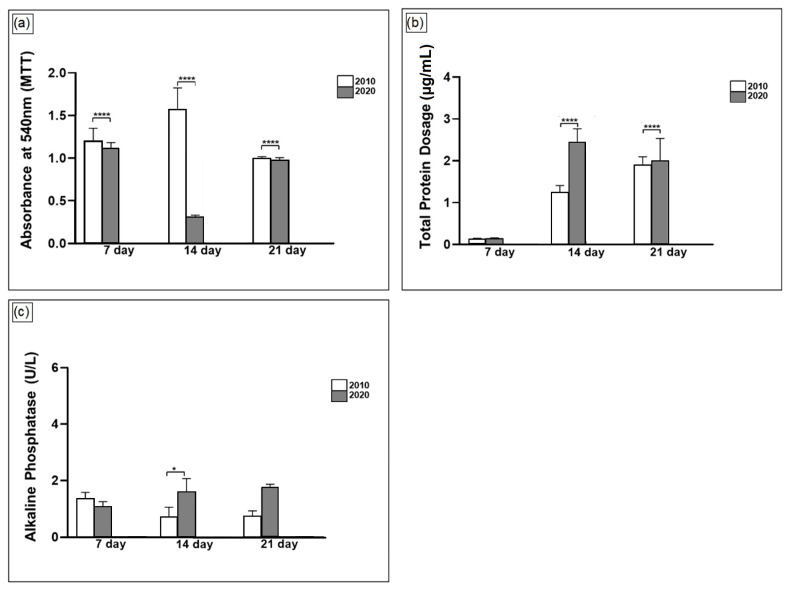
(**a**) MTT activity of MC3T3-E1 cells cultured on PLA/PEG/CaP^1.1^ composite scaffolds for 7, 14, and 21 days. (**b**) Total protein content (µg / mL) at 7, 14 and 21 days of cell culture of the osteoblastic lineage MC3T3 cultured on PLA/PEG/CaP composite scaffolds. (**c**) Levels of ALP activity of MC3T3-E1 cells on PLA/PEG/CaP composite scaffolds after different culture periods (days 7, 14 and 21). The symbols * and **** mark the statistically significant differences between the groups: *p* < 0.05 and *p* < 0.001, respectively.

Once the highest apatite deposition occurred for scaffolds 2010CaP^1.1^ and 2020CaP^1.1^, they were selected to perform MTT, total protein dosage, and ALP tests. Figure 10a shows the viability measurements of MC3T3 cells grown in different samples with PLA/PEG/CaP^1.1^ fiber scaffolds. After 7 days, the cells plated on the scaffolds showed the same viability profile. The major period of differentiation of the osteoblast lineage occurred 14 days after cells were seeded. The best results were observed with the scaffold samples 2010CaP^1.1^, but after 21 days there was equivalence between all samples, demonstrating that all tested scaffolds are good candidates for use in tissue engineering.

The spectrophotometer analysis, at a wavelength of 660 nm, was performed after 7, 14, and 21 days. Figure 10b illustrates how the scaffolds increase the total protein production, referring to both cell proliferation and the bone matrix produced. Therefore, a progressive and consistent increase in total protein production is observed in all samples.

Alkaline phosphatase (ALP) activity is a common marker for the measurement of bone formation and was utilized in this work for the detection of osteoblast differentiation after 7, 14, and 21 days of culture. It was found that the ALP activity of MC3T3-E1 cells cultured on the tested scaffolds was higher with the increasing culture time. This is expected because ALP is generally considered an early biomarker for osteoblastic differentiation of pre-osteoblasts [88]. A higher ALP activity indicates a more advanced stage of differentiation. Low ALP activity could be detected after 7 days of culture, but no significant difference in the ALP activity was observed between composite scaffolds (*p* ˂ 0.05). Fourteen days post-seeding, the MC3T3-E1 cells grown on 2020CaP^1.1^ composite scaffolds exhibited a much higher level of ALP activity than on the other scaffolds (*p* ˂ 0.05), possibly due to the presence of CaP, which is widely known as an inducer of in vitro osteoblastic differentiation of primitive cells towards osteoblasts [89,90,91].

Moreover, this study indicates that the two-step route used produced hybrid systems that could support the osteogenic differentiation of pre-osteoblastic MC3T3-E1 cells into mature osteoblasts and that two-step SBS is a facile route to develop PLA systems incorporated with nanostructured CaPs with potential application in bone repair and regeneration.

## 4. Conclusions

Hybrid 3D nanofibrous scaffolds of PLA/PEG/CaP were rapidly produced by a novel method: using a two-step solution blow spinning (SBS) technique. The amount of CaP (10 and 20%wt) and the Ca/P ratio (1.1 and 1.63) introduced significant effects on the morphology and biological properties of the hybrid materials. The hybrid scaffolds’ mean fiber diameters ranged from 408 ± 141 nm to 893 ± 496 nm, with a randomly interconnected and highly porous structure, besides good dispersion of CaP in the fiber. Incorporation of CaPs and PEG at 20%wt promoted deposition of apatite on the fiber surface. The mixture of β-TCP and HAp (Ca/P = 1.1) in the fibers improved the mineralization process, since the former dissolves in a fast way. The designed hybrid scaffolds can support osteogenic differentiation of pre-osteoblastic MC3T3-E1 cells into mature osteoblasts and can be considered a promising material for applications in bone tissue engineering.

## Figures and Tables

**Table 1:** Scaffold formulation designed for this work.

Nomenclature ^a^	PEG (wt%) ^b^	CaP (wt%) ^b^
PLA20PEG	20	-
2010CaP^1.1^	20	10
2020CaP^1.1^	20	20
2010CaP^1.67^	20	10
2020CaP^1.67^	20	20

^a^ The superscript stands for Ca/P ratio of 1.67 or 1.1; ^b^ relative to PLA weight.

**Table 2:** Mean diameter of PLA/PEG/CaP hybrid scaffolds produced by SBS.

Nomenclature	Mean Diameter ± Standard Deviation (nm)
PLA20PEG	335 ± 121
2010CaP^1.1^	484 ± 291
2020CaP^1.1^	751 ± 350
2010CaP^1.67^	893 ± 496
2020CaP^1.67^	408 ± 141

## Data Availability

Data are contained within the article.
